# Exuberant case of verrucous cutaneous leishmaniasis^☆^

**DOI:** 10.1016/j.abd.2020.11.011

**Published:** 2021-11-26

**Authors:** Jana Regis Novaes, Luise Ribeiro Daltro, Thiago Amparo Ferreira, Paulo Roberto Lima Machado

**Affiliations:** Hospital Universitário Professor Edgard Santos, Universidade Federal da Bahia, Salvador, BA, Brazil

**Keywords:** *Leishmania braziliensis*, Leishmaniasis, cutaneous, Neglected diseases

## Abstract

Cutaneous leishmaniasis represents a public health problem that affects 85 countries. It is an endemic disease in Brazil, having an important socioeconomic impact. An exuberant case of cutaneous leishmaniasis is reported herein. A 28-year-old male patient with Down syndrome had had verrucous plaques on the back for over a year, with progressive growth. PCR of a lesion sample was positive for *Leishmania braziliensis*. The patient's condition was classified as atypical cutaneous leishmaniasis. He was successfully treated with amphotericin B and miltefosine. The treatment remains a challenge, given the toxicity and low cure rate of the currently recommended drugs.

## Case report

**Figure 1:**
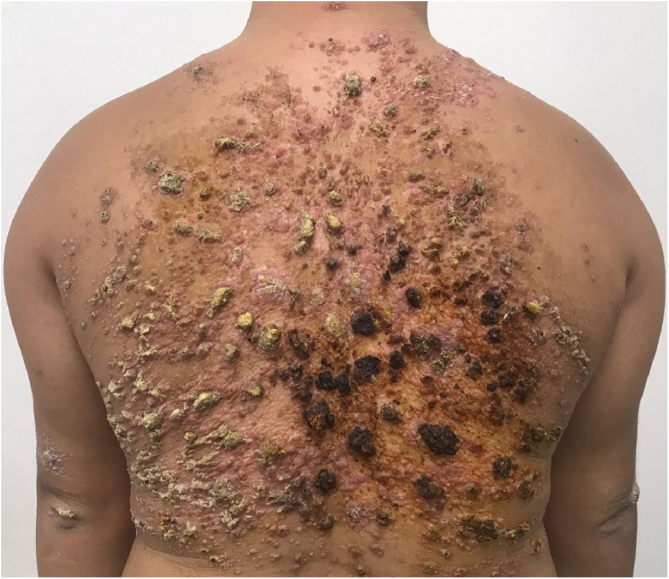
Coalescent papules forming an extensive verrucous plaque on the back, as seen in the first medical consultation.

A 28-year-old male with Down syndrome reported the onset of lesions on his back one year and three months before, with progressive increase (Fig. 1). There was no pruritus, pain, or associated systemic symptoms. He reported previous treatment with itraconazole and terbinafine, with no response. The physical examination disclosed the presence of coalescent papules forming an extensive verrucous plaque on the back, with prominent hyperkeratotic areas, in addition to crusted and eroded lesions with an erythematous base. He also had similar smaller lesions on his arms and left thigh. There were no lesions in the nostrils, oral cavity, or lymph node enlargement.

**Figure 2:**
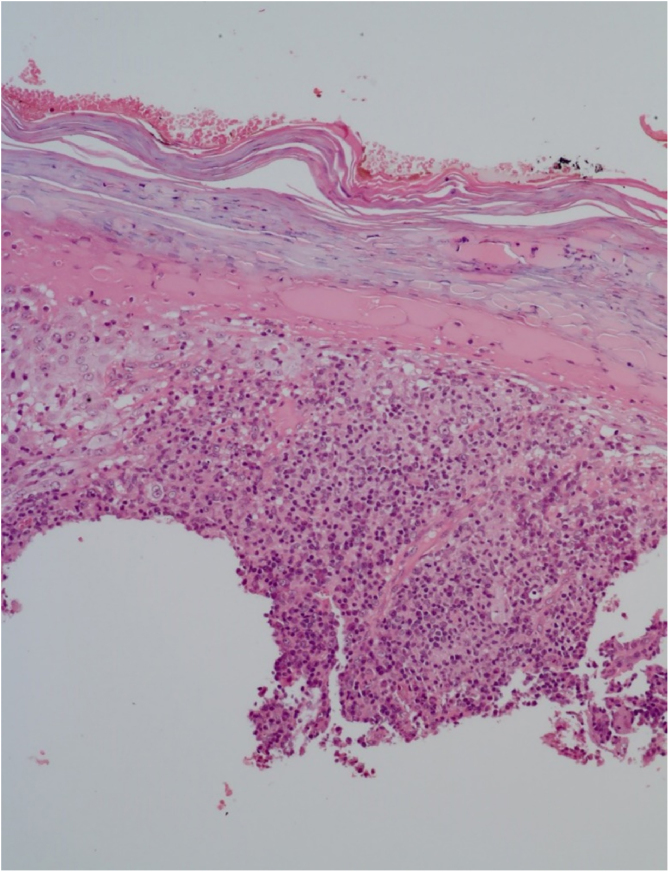
Hyperkeratosis an extensive parakeratosis over a dense lymphohistioplasmocytic inflammatory infiltrate in the dermis, outlining granulomas and numerous neutrophils (Hematoxylin & eosin, ×10).

**Figure 3:**
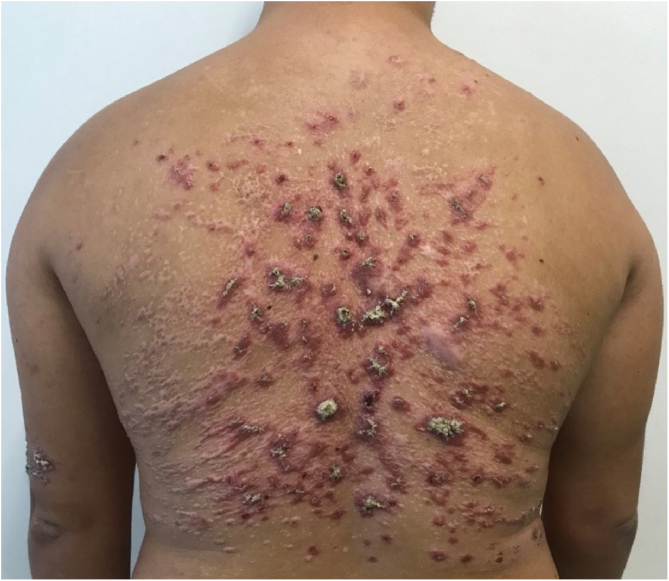
Predominance of scarring lesions after treatment with liposomal Amphotericin B, but still maintaining some active lesions (total accumulated dose of 3g).

He had been previously submitted to an anatomopathological examination at another service whose report showed epidermis with acanthosis, hyper and parakeratosis, dense lymphohistioplasmocytic inflammatory infiltrate in the dermis, outlining granulomas and numerous neutrophils (Fig. 2). Upon the patient’s admission, the PCR of a lesion sample was requested, with a positive result for *Leishmania braziliensis* and a positive Montenegro test (6 mm). The other serologies and complementary tests showed no abnormalities, except for subclinical hypothyroidism and dyslipidemia. The patient was admitted for treatment with amphotericin B deoxycholate (total dose of 1.8 g) due to the extension and exuberance of the skin lesions, with partial improvement of the lesions during follow-up (Fig. 3). While hospitalized, the patient had an increase in nitrogenous slag (urea and creatinine) and a bloodstream infection. *S. aureus* was isolated in blood cultures and he was treated with oxacycline. After three months, he underwent a new course of treatment, this time with liposomal amphotericin B (3g total dose), showing significant improvement of the dermatological condition.

**Figure 4:**
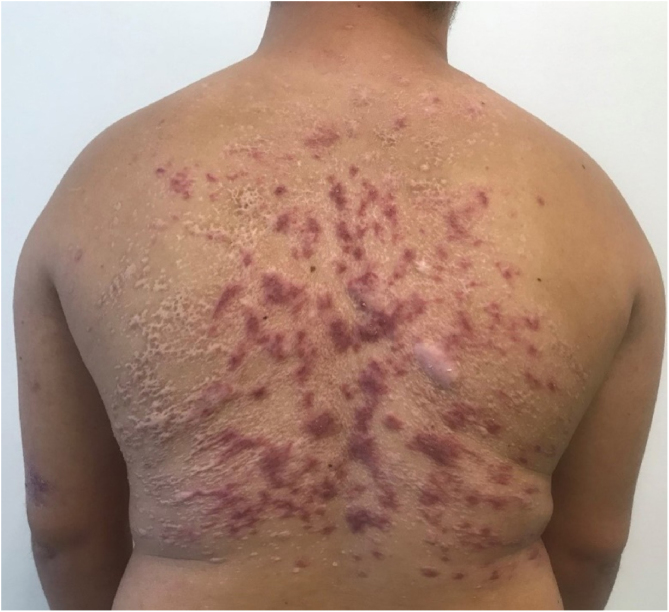
Satisfactory response immediately after treatment with miltefosine.

**Figure 5:**
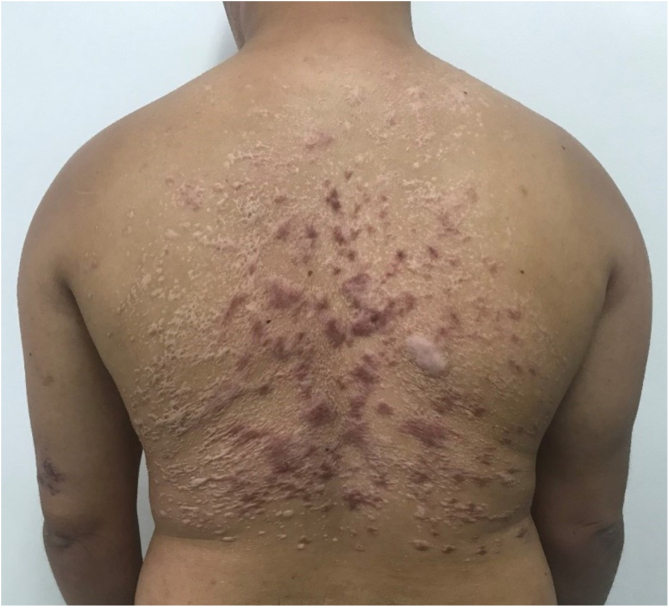
Maintenance of scarring lesions only, three months after completing treatment with miltefosine.

Two months after the second treatment, the patient developed partial recurrence and in an attempt to prevent another prolonged hospitalization with multiple morbidities, treatment with miltefosine 50 mg every 8 hours, for 38 days, was chosen, with good response and tolerability (Fig. 4). The patient maintained a good response at follow-up three months later with only residulal scars (Fig. 5).

## Discussion

American cutaneous leishmaniasis (ACL) is a public health problem that affects 85 countries, with an annual record of approximately one million new cases per year worldwide. In Brazil, there are records of cases in all states.^1^ Transmission to humans occurs through the bite of female sandflies, especially of the genus *Lutzomyia*. ACL is a spectral disease, whose clinical presentation varies according to the parasite species and the host's immune response. The main clinical forms that have been described are the cutaneous, mucosal, disseminated, or diffuse anergic cutaneous form, with highly variable clinical presentations, which can generate disfiguring lesions and definitive deformities.^1^, ^2^ Disseminated leishmaniasis is characterized by lesion polymorphism, which can include acneiform papules, nodules, or ulcers, with at least 10 lesions in at least two non-contiguous body segments, with mucosal involvement in most cases.^2^ These disseminated lesions usually appear two to six weeks after the initial ulcerated lesion, and the association with fever, chills, asthenia, and nausea is frequent.^2^ The main differential diagnoses are diffuse anergic cutaneous leishmaniasis, in which the lesions are more infiltrated and show no ulcerations or mucosal involvement, and cutaneous leishmaniasis with multiple lesions in immunosuppressed patients.^2^

The patient did not develop systemic manifestations and did not have the classical lesions of disseminated or diffuse anergic leishmaniasis either, thus he was classified as atypical cutaneous leishmaniasis, with multiple lesions and a verrucous presentation. Despite the exuberance of the lesions and the patient’s origin from an endemic area, it took more than one year for the patient to undergo adequate investigation and treatment, which highlights the need to alert the population and health professionals to the importance of an early diagnosis. Atypical forms of ACL have become increasingly frequent, particularly in endemic areas.^3^ Moreover, patients with Down syndrome can have innate and adaptive immune response abnormalities, which could explain the rare presentation of the case reported herein.^4^

The pentavalent antimony (meglumine antimoniate) is the first choice for treatment of ACL in Brazil.^1^, ^2^, ^3^, ^4^, ^5^ However, in patients with atypical, severe cases or in those with more than 20 lesions, amphotericin B is indicated, due to the inadequate response to antimonials in these cases.^1^, ^3^ The deoxycholate form is more accessible because it has a lower cost, but it has more adverse effects – renal function impairment, phlebitis, electrolyte disturbances, gastrointestinal symptoms. Liposomal amphotericin B is usually better tolerated.^1^, ^3^

ACL is considered one of the priority neglected diseases, and despite the great advances in medicine in recent years, the treatment of this disease remains a challenge for dermatologists, due to the high toxicity of the available drugs and low cure rates, often requiring multiple treatment cycles to achieve a satisfactory outcome.^2^ Miltefosine may be a promising therapeutic option, as it is administered orally and shows good efficacy and safety.^2^, ^5^

It is extremely important to carry out more studies research on new drugs to treat this disease and many other neglected diseases, aiming to reduce deformities, complications, and the impact on the quality of life of the affected patients.

## Financial support

None declared.

## Authors' contributions

Jana Regis Novaes: Drafting and editing of the manuscript; intellectual participation in the conduct of the case; review of the literature.

Luise Ribeiro Daltro: Drafting and editing of the manuscript; intellectual participation in the conduct of the case; review of the manuscript.

Thiago Amparo Ferreira: Intellectual participation in the conduct of the case; review of the manuscript.

Paulo Roberto Lima Machado: Intellectual participation in the conduct of the case; review of the literature and review of the manuscript.

## Conflicts of interest

None declared.
